# Long‐Term Outcomes Following Achievement of Clinically Inactive Disease in Juvenile Idiopathic Arthritis: The Importance of Definition

**DOI:** 10.1002/art.40519

**Published:** 2018-07-22

**Authors:** Stephanie J. W. Shoop‐Worrall, Suzanne M. M. Verstappen, Janet E. McDonagh, Eileen Baildam, Alice Chieng, Joyce Davidson, Helen Foster, Yiannis Ioannou, Flora McErlane, Lucy R. Wedderburn, W. Thomson, Kimme L. Hyrich

**Affiliations:** ^1^ The University of Manchester, Central Manchester University Hospitals NHS Foundation Trust, Manchester Academic Health Science Centre Manchester UK; ^2^ Alder Hey Children's NHS Foundation Trust Liverpool UK; ^3^ Royal Manchester Children's Hospital Manchester UK; ^4^ The Royal Hospital for Children, Glasgow, UK Edinburgh UK; ^5^ The Royal Hospital for Sick Children Edinburgh UK; ^6^ Great North Children's Hospital, Newcastle Hospitals NHS Foundation Trust Newcastle‐upon‐Tyne UK; ^7^ Newcastle University Newcastle‐upon‐Tyne UK; ^8^ University College London London UK; ^9^ Great North Children's Hospital Newcastle Hospitals NHS Foundation Trust Newcastle‐upon‐Tyne UK; ^10^ University College London, Great Ormond Street Hospital NHS Foundation Trust London UK; ^11^ NIHR Great Ormond Street Hospital Biomedical Research Centre London UK

## Abstract

**Objective:**

Potential targets for treat‐to‐target strategies in juvenile idiopathic arthritis are minimal disease activity (MDA) and clinically inactive disease (CID). We undertook this study to compare short‐ and long‐term outcomes following achievement of MDA and CID on the 10‐joint clinical Juvenile Arthritis Disease Activity Score (cJADAS10) and following achievement of CID on Wallace et al's preliminary criteria.

**Methods:**

Children recruited to the Childhood Arthritis Prospective Study, a UK multicenter inception cohort, were selected if they were recruited prior to January 2011 and diagnosed as having oligoarthritis or rheumatoid factor–negative or –positive polyarthritis. One year following diagnosis, children were assessed for MDA on the cJADAS10 and for CID on both Wallace et al's preliminary criteria and the cJADAS10. Associations were tested between those disease states and functional ability, absence of joints with limited range of motion, psychosocial health, and pain at 1 year and annually to 5 years.

**Results:**

Of 832 children, 70% were female and the majority had oligoarthritis (68%). At 1 year, 21% had achieved CID according to both definitions, 7% according to Wallace et al's preliminary criteria alone, and 16% according to the cJADAS10 alone; 56% had not achieved CID. Only 10% of children in the entire cohort achieved MDA without also achieving CID. Achieving either early CID state was associated with a greater absence of joints with limited range of motion. However, only CID according to the cJADAS10 was associated with improved functional ability and psychosocial health. Achieving CID was superior to achieving MDA in terms of short‐ and long‐term pain and the absence of joints with limited range of motion.

**Conclusion:**

CID on the cJADAS10 may be preferable as a treatment target to CID on Wallace et al's preliminary criteria in terms of both feasibility of application and long‐term outcomes.

Despite the licensing of biologic therapies for juvenile idiopathic arthritis (JIA) 1 and increasingly aggressive treatment strategies 2, a recent systematic review estimates that the burden of disease in JIA remains high, with fewer than 50% of patients achieving remission after a decade of disease 3. Following the success of treat‐to‐target approaches in adult rheumatology 4, 5, a similar approach in JIA may yield better disease outcomes 6. However, it is less clear what the target should be. One target for children and young people with JIA is clinically inactive disease (CID), a state in which no evidence of disease activity is apparent 7. While a state of CID, and ultimately disease remission, would be ideal, it may not be feasible in all children due to the nature of their JIA disease activity. In addition, the treatment required for such a state may not be acceptable when weighed against additional risks of adverse events and the cost of additional therapies. An alternative target could therefore be minimal disease activity (MDA), a state that would include not only children with CID but also those with low but persistent disease activity 8.

Defining disease states such as CID in clinical practice can be challenging and currently relies on composite criteria 3, 9. Multiple such definitions have been proposed, including CID using Wallace et al's preliminary criteria 7, the American College of Rheumatology (ACR) 2011 CID criteria 10, and scoring below certain cutoffs on the Juvenile Arthritis Disease Activity Score (JADAS) 11 or clinical JADAS (cJADAS) 12. Wallace et al's preliminary criteria include 5 components, observed or measured by a physician, all of which must be absent or in the normal range; these components do not include an assessment by the patient or the patient's proxy 7. In contrast, the JADAS and cJADAS include fewer overall components, meaning that they may be easier to complete in a routine clinical setting. They do include the patient's assessment or a proxy's subjective assessment of the patient's well‐being 11, 12. Although Wallace et al's preliminary criteria and low score cutoffs on the JADAS or cJADAS are intended to identify similar disease constructs, a recent analysis has shown that these definitions will classify different groups of children as having CID, which may be driven by their different components 9. It is currently unclear which definition, if any, should be applied in the clinical setting as a treatment target, but the choice may be influenced by how achievement of CID according to each definition relates to later disease outcomes. It is also unclear whether applying increasingly aggressive treatment strategies to achieve CID beyond MDA is favorable in terms of long‐term outcomes.

Therefore, the aims of this study were 1) to describe the impact of early achievement of CID on functional ability, joint limitations, and psychosocial health over the first 5 years following initial presentation to a pediatric rheumatology clinic, 2) to assess whether the applied definition of CID at 1 year is associated with different long‐term outcomes, and 3) to assess whether achieving CID is beneficial beyond MDA in terms of pain in addition to these outcomes according to the 10‐joint cJADAS (cJADAS10).

## Patients And Methods

**Study population.** This analysis included children recruited to the Childhood Arthritis Prospective Study (CAPS), a prospective inception cohort recruiting from 8 UK pediatric and adolescent rheumatology centers since 2001. Details of this cohort have been described previously 13. The CAPS was approved by the Northwest Multicentre Research Ethics Committee, and written informed consent from guardians (and, where appropriate, assent or consent from participants) was obtained.

For this study, children were included if they had a physician's diagnosis of JIA (oligoarticular and either rheumatoid factor [RF]–negative or –positive polyarticular categories) and had been recruited to the CAPS prior to January 1, 2011 to allow for at least 5 years of follow‐up. Children were included in each analysis if outcome data were available for at least one of the time points studied. Those who had not returned study forms after initial presentation were excluded.

**Data collection.** CAPS data were collected from the medical case notes at first presentation to a pediatric rheumatology clinic (baseline date) and annually thereafter for 5 years using a predefined study schedule. These include demographic and disease features, International League of Associations for Rheumatology (ILAR) category 14 as recorded by the treating physician in the case notes, and any antirheumatic treatments. Collection of components of the CID/MDA criteria has been described previously 9.

At each follow‐up visit, proxies (or the children themselves where possible if they were age >11 years) were asked to complete a series of patient‐reported outcome measures, including the Childhood Health Assessment Questionnaire (C‐HAQ) and the Child Health Questionnaire (CHQ) 15. The C‐HAQ score totals 24 and is divided so that final scores range from 0 to 3, with higher scores denoting poorer functional ability. It is known to have a floor effect, whereby scores tend to cluster at the “good functional ability” section of the scale 15. The CHQ is a generic health‐related quality of life (HRQoL) measure designed for proxy completion for pediatric patients age >5 years. It comprises 15 subscales, 10 of which can be aggregated to yield a psychosocial summary score. This summary score ranges from 0 to 100, with higher scores denoting better HRQoL 16. Scores below 30 are considered at least 2 standard deviations below population averages 17. Patients/proxies also completed a 100‐mm pain visual analog scale (VAS).

**States of CID and MDA.** Using data from 1 year following initial presentation, children were categorized according to their CID status on Wallace et al's preliminary criteria 7 and the cJADAS10 12. Children were therefore classified as having the following states: 1) CID on both criteria sets, 2) CID on Wallace et al's preliminary criteria only, 3) CID on the cJADAS10 only, 4) no CID. Children were also classified as to whether they fulfilled: 1) CID on the cJADAS10, 2) MDA but not CID on the cJADAS10 (Table 1).

**Table 1:** Definitions of CID and MDA applied to the Childhood Arthritis Prospective Study cohort^a^

Definition	Components						How to calculate
	No. of joints with active disease	Physician's global assessment	Parent's global assessment	ESR/CRP	Uveitis	Systemic features in systemic JIA	
CID according to Wallace et al's preliminary criteria 7	Yes	Yes	No	Yes	Yes	Yes	Zero or normal across all components
CID according to cJADAS10 12	Yes	Yes	Yes	No	No	No	Total score ≤1
MDA according to cJADAS10 12	Yes	Yes	Yes	No	No	No	For oligoarticular course, score ≤1.5; for polyarticular course, score ≤2.5

a CID = clinically inactive disease; MDA = minimal disease activity; ESR = erythrocyte sedimentation rate; CRP = C‐reactive protein; JIA = juvenile idiopathic arthritis; cJADAS10 = 10‐joint clinical Juvenile Arthritis Disease Activity Score.

**Outcome assessment.** The following outcomes were selected: functional ability on the C‐HAQ, no joints with limited range of motion, psychosocial health on the CHQ, and pain. These outcomes were selected to avoid circular reasoning. For example, the cJADAS10 includes the proxy global assessment of well‐being and Wallace et al's preliminary criteria do not. It would therefore be expected that CID on the cJADAS10 would be more strongly associated with better longitudinal proxy‐assessed well‐being than would CID on Wallace et al's preliminary criteria. To avoid this circular reasoning, all outcomes selected for the current study must not have formed one of the components of either CID/MDA criteria set. All outcomes were assessed annually from baseline to 5 years following initial presentation to a pediatric rheumatology clinic.

**Statistical analysis.** First, associations were tested between 1‐year CID/MDA states cross‐sectionally with the outcomes at 1 year. Second, associations between these 1‐year states and outcomes annually from 1 to 5 years following initial presentation were analyzed via multilevel, multivariable regression analyses. All children with outcomes available for at least one time point were included. Depending on the outcome, the following regression analyses were applied: logistic (no joints with limited range of motion versus any joints with limited range of motion, CHQ psychosocial score <30 versus CHQ psychosocial score ≥30) and linear (CHQ psychosocial score, pain) regressions. The known floor effect of the C‐HAQ, whereby scores cluster at the “high functional ability” end of the scale 15, prompted its analysis using zero‐inflated negative binomial regression models. These models incorporate the excessive zero counts by first generating odds ratios (ORs) for having a score of zero versus not. Second, they produce risk ratios for increasing counts along the C‐HAQ scale among those subjects who have not scored zero. To analyze the C‐HAQ in this way, each value must be an integer. C‐HAQ scores were therefore multiplied by 8 to yield their original score out of 24 points to allow its analysis as a count variable. Because one component of the cJADAS10 criteria set, the parental global assessment of well‐being, has been reported to be driven by pain 18, 19, 20, pain was only used as an outcome when analyzing associations between early CID and MDA on the cJADAS10.

Data were analyzed following multiple imputation under assumptions detailed in previous work 9 for CID/MDA states and under the assumption of data “missing at random” for outcome data except CHQ psychosocial scores. Twenty imputed data sets were generated using Stata software, version 14 (StataCorp), and estimates from individual models were pooled using Rubin's Rules, where both within‐ and across‐imputation variances are accounted for 21. CHQ psychosocial scores were not imputed due to the likely unmeasured confounders that would inform these data.

Random effects were included at the patient level for longitudinal models. The zero‐inflated longitudinal models instead incorporated robust clusters at the patient level. Multivariable models were adjusted for hospital, age, symptom duration and year of presentation, sex, and ILAR category, and models at 1 year were also adjusted for respective outcome at baseline. Covariate multicollinearity was assessed via Spearman's correlations, and zero‐inflated negative binomial models were deemed preferable to Poisson models if dispersion parameter 95% confidence intervals (95% CIs) did not contain zero. All analyses were completed using Stata software, version 14.

## Results

**Patient cohort.** A total of 1,106 patients had been recruited to the CAPS by January 1, 2011. Of these, 274 were excluded (60 were diagnosed as having a non‐JIA condition, 209 did not have oligoarticular or polyarticular JIA, and 5 had not returned study forms). This left 832 patients for the current analyses, including 649 for whom we had available data on the CHQ psychosocial score at any time point (601 from 1 year onward). By the end of the 5‐year follow‐up period, 510 children (61%) remained under care of pediatric rheumatologists and had not been lost to follow‐up or discharged (see Supplementary Figure 1, available on the *Arthritis & Rheumatology* web site at http://onlinelibrary.wiley.com/doi/10.1002/art.40519/abstract). The numbers of children with available data for each outcome across time points are shown in Supplementary Table 1, http://onlinelibrary.wiley.com/doi/10.1002/art.40519/abstract.

Within the cohort, median age at initial presentation to a pediatric rheumatology clinic was 6.9 years (interquartile range [IQR] 3.1–11 years), and median symptom duration at presentation was 5.5 months (IQR 2.9–11 months). Seventy percent of the cohort were females, and 68%, 28%, and 5% were diagnosed as having oligoarticular JIA, RF‐negative polyarticular JIA, and RF‐positive polyarticular JIA, respectively (Table 2).

**Table 2:** Baseline characteristics of the patients in the Childhood Arthritis Prospective Study cohort^a^

	Patients with available baseline data, no. (%)	Median (IQR) or no. (%)	Patients with available data at 1 year, no. (%)	Median (IQR) or no. (%)
Patient characteristics				
Female	832 (100)	586 (70)	–	–
White or Caucasian	832 (100)	752 (90)	–	–
Age at onset, years	827 (99)	5.9 (2.4–9.9)	–	–
Age at first presentation, years	832 (100)	6.9 (3.1–11)	–	–
Symptom duration at diagnosis, months	827 (99)	5.5 (2.9–11)	–	–
ILAR category	832 (100)		–	–
Oligoarticular		563 (68)	–	–
RF− polyarticular		231 (28)	–	–
RF+ polyarticular		38 (5)	–	–
Disease characteristics				
No. of joints with active disease (78 joints assessed)	784 (94)	2 (1–5)	689 (83)	0 (0–1)
No. of joints with limited range of motion (78 joints assessed)	784 (94)	1 (1–3)	677 (81)	0 (0–1)
No joints with limited range of motion	784 (94)	161 (21)	677 (81)	391 (58)
PGA, 0–10‐cm VAS	630 (76)	2.8 (1.5–5.0)	576 (69)	0.4 (0.0–1.8)
PGE, 0–10‐cm VAS	546 (66)	2.1 (0.5–5.0)	587 (71)	0.6 (0.0–2.5)
ESR, mm/hour	517 (62)	16 (6–40)	194 (23)	8 (4–17)
CRP, mg/liter	474 (57)	7 (4–19)	173 (21)	4 (3–7)
Uveitis	644 (77)	27 (4.2)	673 (81)	30 (4.5)
C‐HAQ score, 0–3	557 (67)	0.8 (0.1–1.4)	573 (69)	0.3 (0–0.9)
Pain, 0–100‐mm VAS	552 (66)	30 (8–58)	572 (69)	8 (1–33)
CHQ score, 0–100	281 (34)	50 (39–55)	343 (41)	52 (43–58)
CHQ score ≤30	281 (34)	32 (11)	343 (41)	23 (6.7)

a IQR = interquartile range; ILAR = International League of Associations for Rheumatology; RF = rheumatoid factor; PGA = physician's global assessment of disease activity; VAS = visual analog scale; PGE = proxy's global assessment of well‐being; ESR = erythrocyte sedimentation rate; CRP = C‐reactive protein; C‐HAQ = Childhood Health Assessment Questionnaire; CHQ = Child Health Questionnaire.

**Frequency of CID and MDA among patients at 1 year.** One year following initial presentation, the majority of patients had not achieved CID (56%). Twenty‐one percent had achieved both CID states, and an additional 23% had achieved only one state of CID (16% on the cJADAS10 and 7% on Wallace et al's preliminary criteria). On the cJADAS10, 48% of patients had achieved MDA. Of those patients, 79% had also achieved CID (38% of the entire cohort).

**Association between early achievement of CID and outcomes measured at 1 year.** All estimates from complete case analyses were similar to those following multiple imputation (see Supplementary Tables 2 and 3, http://onlinelibrary.wiley.com/doi/10.1002/art.40519/abstract). The following results relate to imputed data, except for CHQ psychosocial scores. All models met the tested assumptions.

At 1 year, achievement of any state of CID was associated with significantly increased odds of having no joints with limited range of motion (for Wallace et al's preliminary criteria only, OR 7.5 [95% CI 2.9, 19.2]; for cJADAS10 only, OR 3.9 [95% CI 2.5, 6.3]; for both criteria sets, OR 9.3 [95% CI 4.9, 17.7]). However, children who had achieved CID only on Wallace et al's preliminary criteria but not on the cJADAS10 had no better CHQ psychosocial scores or C‐HAQ scores than those with active disease at 1 year. In contrast, those children who had achieved CID on at least the cJADAS10 scored at least 5 points better on CHQ psychosocial health (for cJADAS10 only, coefficient 5.3 [95% CI 0.5, 10.1]; for both cJADAS10 and Wallace et al's preliminary criteria, coefficient 5.5 [95% CI 1.5, 9.4]) than did children with active disease. Those children also had at least 4 times the odds of having no disability recorded using the C‐HAQ (for cJADAS10 only, OR 4.5 [95% CI 2.2, 9.5]; for both cJADAS10 and Wallace et al's preliminary criteria, OR 5.2 [95% CI 2.7, 9.9]) than did those with active disease. When assessing nonzero C‐HAQ scores, children who had achieved CID on the cJADAS10 had 50% lower scores (for cJADAS10 only, 95% CI 20%, 60%; for both cJADAS10 and Wallace et al's preliminary criteria, 95% CI 30%, 70%) (Table 3). Too few children who had achieved CID on either criteria set scored <30 on CHQ psychosocial health, so associations with this outcome could not be tested.

**Table 3:** Multivariable associations between disease activity and outcomes 1 year after initial presentation to a pediatric rheumatology clinic^a^

Disease state 1 year after presentation	OR for C‐HAQ score = 0 (95% CI)	*P*	IRR of higher C‐HAQ score if C‐HAQ score >0 (95% CI)	*P*	OR for no joints with limited range of motion (95% CI)	*P*	Coefficient of higher CHQ psychosocial score (95% CI)	*P*	Coefficient of greater pain, mm (95% CI)	*P*
CID states										
No CID on either tool	Reference	–	Reference	–	Reference	–	Reference	–	–	–
CID on Wallace et al's preliminary criteria only	0.8 (0.3, 3.2)	0.975	1.1 (0.8, 1.5)	0.503	7.5 (2.9, 19.2)	<0.001	3.1 (−3.3, 9.5)	0.335	–	–
CID on cJADAS10 only	4.5 (2.2, 9.5)	<0.001	0.5 (0.4, 0.8)	0.002	3.9 (2.5, 6.3)	<0.001	5.3 (0.5, 10.1)	0.029	–	–
CID on both Wallace et al's preliminary criteria and cJADAS10	5.2 (2.7, 9.9)	<0.001	0.5 (0.3, 0.7)	<0.001	9.3 (4.9, 17.7)	<0.001	5.5 (1.5, 9.4)	0.007	–	–
CID vs. MDA on cJADAS10										
MDA only	Reference	–	Reference	–	Reference	–	Reference	–	Reference	–
CID	2.6 (1.0, 7.2)	0.063	0.8 (0.5, 1.2)	0.265	2.4 (1.3, 4.5)	0.006	−0.3 (−5.3, 4.8)	0.914	−6.5 (−12.1, −0.9)	0.023

a Multivariable models are adjusted for age (years) and disease duration (months) at presentation, sex, hospital, and International League of Associations for Rheumatology category (persistent oligoarthritis, extended oligoarthritis, rheumatoid factor (RF)–negative polyarthritis, and RF‐positive polyarthritis). Clinically inactive disease (CID)/minimal disease activity (MDA) states were imputed under various outcomes (see Patients and Methods), and outcomes were imputed under “missing at random” assumptions except for Child Health Questionnaire (CHQ) scores, which were analyzed under complete case analyses (n = 343). OR = odds ratio; C‐HAQ = Childhood Health Assessment Questionnaire; 95% CI = 95% confidence interval; IRR = incidence rate ratio; cJADAS10 = 10‐joint clinical Juvenile Arthritis Disease Activity Score.

**Association between early MDA versus CID on the cJADAS10 and outcomes measured at 1 year.** Compared with children who met the threshold for MDA on the cJADAS10 but who did not also achieve CID, those who did achieve CID had greater odds of no joints with limited range of motion (OR 2.4 [95% CI 1.3, 4.5]) and lower VAS pain scores (coefficient −6.5 mm [95% CI −12.1 mm, −0.9 mm]) at 1 year. However, there were no significant differences between these 2 groups of children in any of the C‐HAQ or CHQ psychosocial outcomes (Table 3).

**Figure 1:**
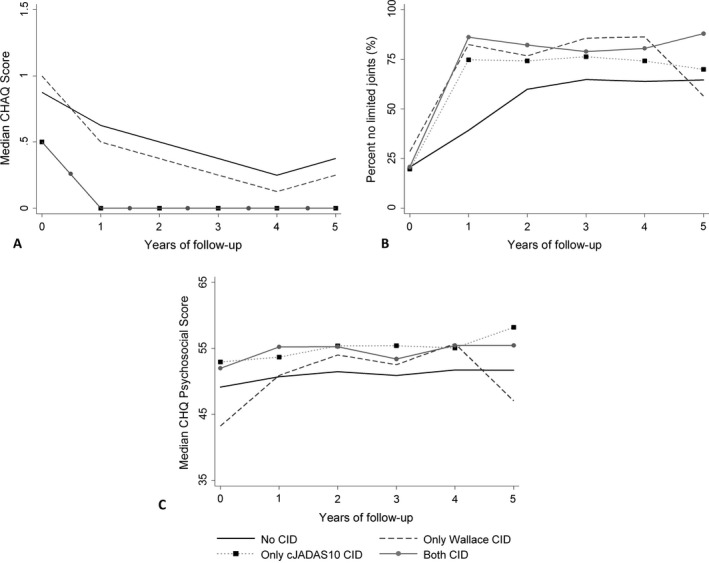
Outcomes over 5 years following initial presentation to a pediatric rheumatology clinic, split according to clinically inactive disease (CID) state at 1 year. **A,** Median Childhood Health Assessment Questionnaire (C‐HAQ) scores. Median C‐HAQ scores over the 5 years were the same whether CID was measured according to both Wallace et al’s preliminary criteria and the 10‐joint clinical Juvenile Arthritis Disease Activity Score (cJADAS10) or according to the cJADAS10 alone. **B,** Percentages of subjects having no joints with limited range of motion. **C,** Median Child Health Questionnaire (CHQ) psychosocial scores.

**Associations between disease activity state at 1 year and long‐term outcomes.** Early achievement of CID on either criteria set was associated with between 2.0 times (for cJADAS10 only, 95% CI 1.5, 2.9) and 3.0 times (for Wallace et al's preliminary criteria only, 95% CI 1.4, 4.5; for both criteria sets, 95% CI 2.0, 4.5) the odds of no joints with limited range of motion for each additional year through 5 years compared with children who had active disease at 1 year (Table 4). Achievement of CID on the cJADAS10 was associated with better CHQ psychosocial scores (for cJADAS10 only, β = 4.1 [95% CI 1.8, 6.4]; for both criteria sets, β = 3.9 [95% CI 1.6, 6.2]) and a higher probability of both no disability and lower disability among those with nonzero C‐HAQ scores compared with those with active disease. There was no difference in long‐term C‐HAQ or CHQ scores between children who had active disease at 1 year and those with CID according to Wallace et al's preliminary criteria but not according to the cJADAS10 (Figure 1). There was no difference across all groups in the proportion of children with CHQ scores <30 (Table 4).

**Table 4:** Multivariable associations between 1‐year disease states and outcomes over the first 5 years after initial presentation^a^

Outcome definition 1 year after presentation	IRR of C‐HAQ score = 0 (95% CI)	*P*	IRR of higher C‐HAQ score if C‐HAQ score >0 (95% CI)	*P*	OR for no joints with limited range of motion (95% CI)	*P*	Coefficient of higher CHQ psychosocial score (95% CI)	*P*	OR for CHQ psychosocial score <30 (95% CI)	*P*	RR for greater pain, mm (95% CI)	*P*
CID states												
No CID on either tool	Reference	–	Reference	–	Reference	–	Reference	–	Reference	–	–	–
CID on Wallace et al's preliminary criteria only	0.8 (0.4, 1.4)	0.364	1.1 (0.9, 1.3)	0.389	2.5 (1.4, 4.5)	0.002	−0.1 (−3.8, 3.5)	0.944	1.8 (0.4, 8.6)	0.469	–	–
CID on cJADAS10 only	2.5 (1.8, 3.6)	<0.001	0.7 (0.6, 0.9)	0.001	2.0 (1.5, 2.9)	<0.001	4.1 (1.8, 6.4)	0.001	0.3 (0.1, 1.2)	0.086	–	–
CID on both Wallace et al's preliminary criteria and cJADAS10	2.5 (1.8, 3.5)	<0.001	0.8 (0.7, 0.9)	0.002	3.0 (2.0, 4.5)	<0.001	3.9 (1.6, 6.2)	0.001	0.3 (0.1, 1.2)	0.085	–	–
CID vs. MDA on cJADAS10												
MDA only	Reference	–	Reference	–	Reference	–	Reference	–	Reference	–	Reference	–
CID	1.5 (0.9, 2.6)	0.113	0.9 (0.8, 1.2)	0.540	1.7 (1.0, 2.7)	0.045	0.6 (−2.6, 3.9)	0.712	1.3 (0.1, 12.6)	0.844	−5.5 (–10.1, –0.9)	0.020

a Multivariable models are adjusted for age (years) and disease duration (months) at presentation, sex, and International League of Associations for Rheumatology category (persistent oligoarthritis, extended oligoarthritis, rheumatoid factor (RF)–negative polyarthritis, and RF‐positive polyarthritis). Missing data for clinically inactive disease (CID)/minimal disease activity (MDA) were imputed under various assumptions (see Patients and Methods), and outcome data were imputed under the assumption of data “missing at random,” except for Child Health Questionnaire (CHQ) scores, which were analyzed using complete case analysis (n = 601). IRR = incidence rate ratio; C‐HAQ = Childhood Health Assessment Questionnaire; 95% CI = 95% confidence interval; OR = odds ratio; RR = risk ratio; cJADAS10 = 10‐joint clinical Juvenile Arthritis Disease Activity Score.

**Figure 2:**
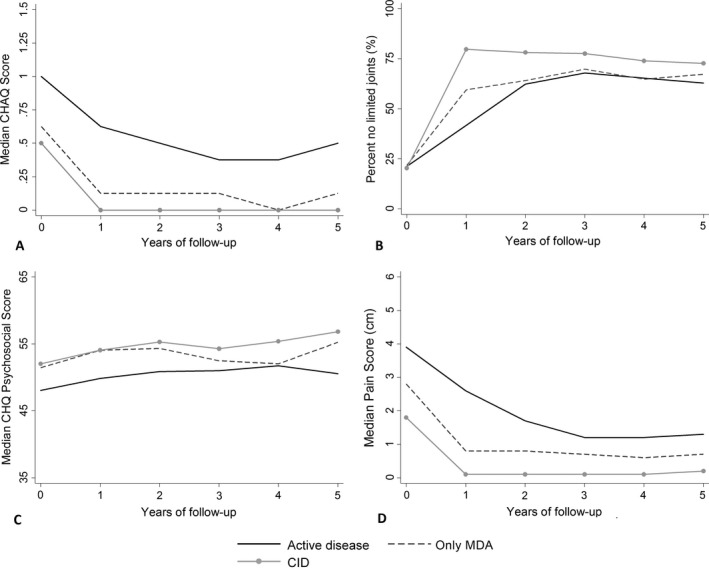
Outcomes over 5 years following initial presentation to a pediatric rheumatology clinic, split according to clinically active disease, clinically inactive disease (CID), and minimal disease activity (MDA) on the 10‐joint clinical Juvenile Arthritis Disease Activity Score (cJADAS10). **A,** Median Childhood Health Assessment Questionnaire (C‐HAQ) scores. **B,** Percentages of subjects having no joints with limited range of motion. **C,** Median Child Health Questionnaire (CHQ) psychosocial scores. **D,** Median pain scores.

**Associations between early MDA versus CID and long‐term outcomes.** Compared with children who had achieved MDA but not CID on the cJADAS10, those who had achieved CID at 1 year had, on average with each increasing year, 1.7 times the odds of no joints with limited range of motion (95% CI 1.0, 2.7) and −5.5 mm better pain scores (95% CI −10.1 mm, −0.9 mm) through 5 years. There was no difference between these patient groups in C‐HAQ or CHQ scores (Table 4 and Figure 2).

## Discussion

The success of treat‐to‐target strategies in adult rheumatology, such as aiming for a low Disease Activity Score in 28 joints 4, 5, 22, has prompted the consideration of similar strategies in pediatric practice 6, 23, 24, 25, 26. One central barrier to implementing treat‐to‐target approaches in JIA is the lack of a single “best” target. Although most would agree that CID is the ultimate target, there are multiple ways in which this disease state can be assessed in the clinical setting. Also important in selecting a “best” outcome measure for clinical practice is understanding how it relates to longer term outcomes. Two such definitions were assessed in this analysis: CID according to Wallace et al's preliminary criteria or according to the cJADAS10. These 2 scores differ in their components. Determination of CID according to Wallace et al's preliminary criteria is limited to assessments by a physician or laboratory measures of inflammation. These criteria also include an assessment of uveitis activity 7. The cJADAS10 captures a lack of inflammation, as assessed by the physician (although with fewer components), but it also includes an assessment by the patient/parent 12. It does not include uveitis activity.

The results of this analysis show that children who achieve CID at 1 year according to either measure have lower counts of joints with limited range of motion both at 1 year and over the next 4 years of follow‐up. However, children who achieved CID according to Wallace et al's preliminary criteria but not according to the cJADAS10 were consistently found to have high levels of disability and poorer psychosocial function. Previous analysis has shown that this difference is driven by lower levels of patient well‐being, despite the absence of joints with active disease or other inflammatory manifestations of disease 9.

This study benefitted from a large sample of patients with JIA in all 3 ILAR categories assessed, all of whom were treated within a single health care service. The scale of the data collected meant that 5‐year outcomes could be assessed following early achievement of different CID states. In addition, robust methods, including imputation methods under clinically plausible assumptions, were implemented to deal with the inevitable missing data associated with observational cohorts. In particular, a large proportion of patients were lost to follow‐up. Informative dropout in the majority of cases informed the imputation methods for CID/MDA states. In turn, this information was used to impute missing outcome values. Thus, although precision of model estimates is affected by missing data, the point estimates should be relatively unbiased.

A challenge is in understanding how best to apply these results in the clinical setting. As achievement of CID according to the cJADAS10 was associated with outcomes equivalent or superior to those for CID achieved according to Wallace et al's preliminary criteria, and since the cJADAS10 is more feasible to complete in clinical practice because it contains only 3 routinely collected components 27, one could argue that this is likely to be a superior treatment target for application in clinical practice. However, a number of limitations of both the outcome measure and the analysis should be considered.

As the 2 scores differ in their components, one could argue that they are not capturing the same construct. Wallace et al's preliminary criteria capture more objective measures of inflammation, while the cJADAS10, through inclusion of a measure of patient well‐being, may also capture other noninflammatory components of the disease, such as chronic pain and fatigue not captured by Wallace et al's preliminary criteria. However, in addition to a single score/cutoff, the value of the individual components of the cJADAS10 would be required to guide individual treatment decisions. Although it is well recognized that functional ability, HRQoL, and pain do improve following treatment with both methotrexate and biologic therapies 28, 29, 30, 31, 32, 33, treating to a cJADAS10 target may also require a multifactorial treatment strategy, potentially including interventions such as physiotherapy and psychological services for children with chronic pain in the absence of joints with active disease. Otherwise, there is the risk of intensifying or changing immunosuppressive therapy in the absence of inflammation. Equally important, relying solely on Wallace et al's preliminary criteria may guide immunosuppressive therapy very well but may ignore other symptoms relevant to the patient.

At the outset of this analysis, it was also unknown whether achievement of CID is associated with better outcomes than those for patients who achieve MDA but not CID. The present study found that achieving CID on the cJADAS10 is associated with fewer joints with limited range of motion compared with achieving MDA. However, achieving CID above MDA was not associated with greater improvements in C‐HAQ or CHQ scores, either between baseline and 1 year or from 1 to 5 years. Therefore, MDA on the cJADAS10 may be an appropriate target when disease activity parameters are low but patient well‐being is poor. The risk of adverse effects with treatment intensification should be considered, particularly if the attainment of CID is deemed unlikely 34 or patient well‐being is high.

A limitation of this study was that, to avoid circular reasoning, important disease activity variables such as counts of joints with active disease could not be used as outcomes. Since the variables differentially form the CID states, any state including said variable would be intrinsically more likely to associate with the outcome. The outcomes selected for the study did, however, comprise multiple physician‐ and patient‐important outcomes. However, these conclusions can only relate to oligoarticular and polyarticular JIA. The CID definitions have only been validated in these categories, and it is likely that additional components will need to be added to these criteria sets in order to fully capture low and inactive disease in less common JIA categories. In addition, due to a lack of data on morning stiffness, we were not able to compare outcomes following the achievement of the 2011 ACR CID criteria with the other CID states. Finally, although it has been suggested that treat‐to‐target strategies will result in better long‐term outcomes, during the period of data collection for this study there was no formal treat‐to‐target strategy in place in the UK. Therefore, although the findings support the notion that early achievement of CID is associated with better outcomes, the data cannot be used to show that active treatment toward these targets currently results in better long‐term outcomes. Further work will need to assess long‐term outcomes following the implementation of these guidelines.

In conclusion, early achievement of CID according to the cJADAS10 is associated with long‐term outcomes equivalent or superior to those associated with early achievement of CID according to Wallace et al's preliminary criteria. Differences in the components of these 2 definitions and the implications for clinical practice through implementation of a single score suggest that the optimal definition of CID for application in a clinical setting remains unclear. Further work, ideally involving consumers, clinicians, and researchers, is needed to best define treatment targets and treatment strategies for use in JIA. The results do, however, highlight the importance of addressing all aspects of JIA and not just the underlying inflammation, in terms of best outcomes for the child.

## Author Contributions

All authors were involved in drafting the article or revising it critically for important intellectual content, and all authors approved the final version to be published. Dr. Hyrich had full access to all of the data in the study and takes responsibility for the integrity of the data and the accuracy of the data analysis.

### Study conception and design

Shoop‐Worrall, Verstappen, Thomson, Hyrich.

### Acquisition of data

Baildam, Chieng, Davidson, Foster, Ioannou, McErlane, Wedderburn, Thomson, Hyrich.

### Analysis and interpretation of data

Shoop‐Worrall, Verstappen, McDonagh, Hyrich.
