# Supplementary material for: Long‐Term Outcomes Following Achievement of Clinically Inactive Disease in Juvenile Idiopathic Arthritis: The Importance of Definition
Source: Arthritis Rheumatol. 2018 Jul 22;70(9):1519–29. doi: 10.1002/art.40519 (PMC6175118; doi:10.1002/art.40519)
Supplement: Supplementary file 1 [file ART-70-1519-s001.docx]

**SUPPLEMENTARY MATERIALS**

**Supplementary Table 1.** Proportion of available of data across outcomes and time points in the study cohort

| Outcome | Total number in analysis  (total n=832) | Data available at each time point (N, %)*^1^ | | | | | |
| --- | --- | --- | --- | --- | --- | --- | --- |
|  |  | Baseline | Year 1 | Year 2 | Year 3 | Year 4 | Year 5 |
| CHAQ | 768 | 554 (72) | 573 (75) | 514 (67) | 465 (61) | 414 (54) | 351 (46) |
| Limited joint count | 827 | 785 (95) | 677 (82) | 568 (69) | 459 (56) | 330 (40) | 224 (27) |
| CHQ psychosocial | 643*^2^ | 281 (44) | 343 (53) | 342 (53) | 356 (55) | 347(54) | 290 (45) |
| Pain | 770 | 553 (72) | 572 (74) | 512 (66) | 461 (60) | 414 (54) | 353 (46) |

*^1^Percent out of children included in the analysis for the corresponding outcome *^2^ 601 children available for longitudinal analysis, 343 used in the analysis at one year. CHAQ: Childhood Health Assessment Questionnaire, CHQ: Child Health Questionnaire

**Supplementary Table 2.** Multivariable associations between disease activity and outcomes at one year following initial presentation to rheumatology under complete case analyses

| Disease state at one year following presentation | OR of CHAQ=0 (95% CI) | P-value | IRR of higher CHAQ if CHAQ >0  (95% CI) | P-value | OR for no limited joints  (95% CI) | P-value | Coef higher CHQ psychosocial  (95% CI) | P-value | Coef greater pain (mm) (95% CI) | P-value |
| --- | --- | --- | --- | --- | --- | --- | --- | --- | --- | --- |
| CID states | | | | | | | | | | |
| Not in CID on either tool | Reference | - | Reference | - | Reference | - | Reference | - | - | - |
| CID Wallace’s preliminary criteria only | 0.7  (0.1, 5.1) | 0.710 | 1.1  (0.7, 1.5) | 0.751 | **7.1**  **(2.7, 18.6)** | **<0.001** | 3.1  (-3.3, 9.5) | 0.335 | - | - |
| CID cJADAS10 only | **4.8**  **(1.3, 16.9)** | **<0.015** | **0.5**  **(0.3, 0.9)** | **0.025** | **4.2**  **(2.6, 6.8)** | **<0.001** | **5.3**  **(0.5, 10.1)** | **0.029** | - | - |
| CID on both Wallace’s preliminary criteria and cJADAS10 | **5.5**  **(2.1, 14.4)** | **0.001** | **0.5**  **(0.3, 0.7)** | **<0.001** | **8.6**  **(4.8, 15.5)** | **<0.001** | **5.5**  **(1.5, 9.4)** | **0.007** | - | - |
| CID vs. MDA on the cJADAS10 | | | | | | | | | | |
| MDA only | Reference |  | Reference |  | Reference |  | Reference |  | Reference |  |
| CID | 3.4  (0.8, 14.2) | 0.088 | 0.7  (0.4, 1.1) | 0.088 | **2.3**  **(1.2, 4.4)** | **0.015** | -0.3  (-5.3, 4.8) | 0.914 | -6.7  (-15.0, 1.6) | 0.113 |

Bold=p<0.05. Multivariable models adjust for age (yrs), disease duration (months) and calendar year at presentation, gender and ILAR subtype (persistent oligo, extended oligo, RF- poly, RF+ poly). Missing CID/MDA states were imputed using a combination of assumptions (see methods) and all outcomes were analysed using complete case analyses. CID: Clinically inactive disease; MDA: Minimal disease activity; cJADAS10: Clinical Juvenile Arthritis Disease Activity Score using a 10 joint count; CHAQ: Childhood Health Assessment Questionnaire; CHQ: Child Health Questionnaire; CI: Confidence interval; IRR: Incidence risk ratio; OR: Odds ratio

**Supplementary Table 3.** Multivariable associations between one year disease states and outcomes over the first five years of disease using complete case analyses

| Outcome definition at 1 year following presentation | IRR of CHAQ=0 (95% CI) | P-value | IRR of higher CHAQ if CHAQ>0  (95% CI) | P-value | OR for no limited joints  (95% CI) | P-value | Coef higher CHQ psychosocial (95% CI) | P-value | OR CHQ psychosocial <30  (95% CI) | P-value | RR greater pain (mm) (95% CI) | P-value |
| --- | --- | --- | --- | --- | --- | --- | --- | --- | --- | --- | --- | --- |
| CID states | | | | | | | | | | | | |
| Not in CID on either tool | Reference | - | Reference | - | Reference | - | Reference | - | Reference | - | - | - |
| CID Wallace’s preliminary criteria only | 0.7  (0.4, 1.4) | 0.301 | 1.1  (0.9, 1.5) | 0.350 | **3.7**  **(1.9, 7.2)** | **<0.001** | -0.1  (-3.8, 3.5) | 0.944 | 1.8  (0.4, 8.6) | 0.469 | - | - |
| CID cJADAS10 only | **3.0**  **(1.9, 4.6)** | **<0.001** | **0.6**  **(0.4, 0.8)** | **0.002** | **2.4**  **(1.7, 3.5)** | **<0.001** | **4.1**  **(1.8, 6.4)** | **0.001** | 0.3  (0.1, 1.2) | 0.086 | - | - |
| CID on both Wallace’s preliminary criteria and cJADAS10 | **3.0**  **(2.0, 4.5)** | **<0.001** | **0.7**  **(0.5, 0.9)** | **0.003** | **4.5**  **(2.8, 7.1)** | **<0.001** | **3.9**  **(1.6, 6.2)** | **0.001** | 0.3  (0.1, 1.2) | 0.085 | - | - |
| CID vs. MDA on the cJADAS10 | | | | | | | | | | | | |
| MDA only | Reference | - | Reference | - | Reference | - | Reference | - | Reference | - | Reference | - |
| CID | 1.6  (0.9, 3.1) | 0.130 | 0.8  (0.6, 1.1) | 0.188 | **2.2**  **(1.3, 3.7)** | **0.003** | 0.6  (-2.6, 3.9) | 0.712 | 1.3  (0.1, 12.6) | 0.844 | **-6.4**  **(-11.4, -1.4)** | **0.012** |

Bold=p<0.05. Multivariable models adjust for age (yrs), disease duration (months) and calendar year at presentation, gender and ILAR subtype (persistent oligo, extended oligo, RF- poly, RF+ poly). Missing CID/MDA states were imputed using a combination of assumptions (see methods) and all outcomes were analysed using complete case analyses. CID: Clinically inactive disease; MDA: Minimal disease activity; cJADAS10: Clinical Juvenile Arthritis Disease Activity Score using a 10 joint count; CHAQ: Childhood Health Assessment Questionnaire; CHQ: Child Health Questionnaire; CI: Confidence interval; IRR: Incidence risk ratio; OR: Odds ratio

| **Baseline**  **Removed:**   - -60 not JIA - -5 no medical case notes - - 209 Not oligo or polyarticular subtypes   Total JIA recruited to CAPS before Jan 2011: **1106**  Total patients with JIA in this analysis: **832** | **Year 1**  **Lost to follow-up:**   - -23 discharged: - 9 well - 4 repeat non-attendance - 8 moved clinic - 1 other   Total at follow-up: **809** | **Year 2**  **Lost to follow-up:**   - -1 skipped follow-up - -37 discharged: - 17 well - 6 repeat non-attendance - 13 moved clinic - 1 other - -4 lost to follow-up   Total at follow-up: **767** | **Year 3**  **Lost to follow-up:**   - -1 skipped follow-up - -49 discharged: - 18 well - 23 moved clinic - 8 repeat non-attendance - -2 lost to follow-up   Total at follow-up: **715** |
| --- | --- | --- | --- |
| **Year 4**  **Lost to follow-up:**   - -12 skipped follow-up - -49 discharged: - 24 well - 9 repeat non-attendance - 16 moved clinic - -28 lost to follow-up   Total at follow-up: **626** | **Year 5**  **Lost to follow-up:**   - -35 discharged: - 19 well - 1 repeat non-attendance - 15 moved clinic - -81 lost to follow-up or skipped follow-up but remained in CAPS   Total at follow-up: **510** |  | |

**Supplementary Figure 1.** Patient flow through the study
